# A Triplex Crystal Digital RT-PCR for the Detection of Avian Leukosis Virus, Chicken Infectious Anemia Virus, and Fowl Adenovirus

**DOI:** 10.3390/ani16142269

**Published:** 2026-07-22

**Authors:** Huayue Zeng, Dandan Hu, Kaichuang Shi, Yu Gan, Yanwen Yin, Feng Long, Shuping Feng, Sujie Qu, Wenjun Lu

**Affiliations:** 1 College of Animal Science and Technology, Guangxi University, Nanning 530005, China; zenghuayve@163.com (H.Z.); hudandan@gxu.edu.cn (D.H.); 2 Guangxi Center for Animal Disease Control and Prevention, Nanning 530001, China; gxgy2025@163.com (Y.G.); yanwen0349@126.com (Y.Y.); longfeng1136@163.com (F.L.); fsp166@163.com (S.F.); mingdao120@126.com (S.Q.); nnlwj@126.com (W.L.)

**Keywords:** avian leukosis virus (ALV), chicken infectious anemia virus (CIAV), fowl adenovirus (FAdV), multiplex reverse transcription–crystal digital PCR (RT-cdPCR)

## Abstract

Avian leukosis virus (ALV), chicken infectious anemia virus (CIAV), and fowl adenovirus (FAdV) are significant immunosuppressive agents that cause huge financial losses to the poultry industry. In this study, specific primers and probes for ALV, CIAV, and FAdV were designed, and the RNA standard for ALV and plasmid standards for CIAV and FAdV were constructed. A triplex reverse transcription–crystal digital PCR (RT-cdPCR) assay was developed for the detection of ALV, CIAV, and FAdV after the optimization of key reaction parameters, including primer and probe concentrations, annealing temperature, and reaction cycle. Subsequently, the assay was assessed for its specificity, sensitivity, and repeatability. The results showed that the assay specifically detected ALV, CIAV, and FAdV, without cross-reactivity with other avian pathogens. The limits of detection (LODs) for sensitivity to ALV, CIAV, and FAdV were 3.75, 3.75, and 5.50 copies/reaction, respectively. Repeatability analysis showed the coefficients of variation (CVs) with 0.38–1.77% for intra-assay and 0.31–3.02% for inter-assay. The assay was applied to evaluate 1211 clinical samples from Guangxi Zhuang Autonomous Region in China and yielded positivity rates of 45.58% (552/1211) for ALV, 10.73% (130/1211) for CIAV, and 5.20% (63/1211) for FAdV. Additionally, these 1211 clinical samples were tested using the reference methods, and the results were compared with those of the established method, showing coincidence rates ≥ 98.18%. In conclusion, a triplex RT-cdPCR was successfully established for the simultaneous and accurate detection of ALV, CIAV, and FAdV.

## Simple Summary

Avian leukosis virus (ALV), chicken infectious anemia virus (CIAV), and fowl adenovirus (FAdV) are important pathogens that induce various types of tumors and immunosuppression and cause huge economic losses to the poultry industry. In this study, a triplex reverse transcription–crystal digital PCR (RT-cdPCR) assay was developed for the detection of ALV, CIAV, and FAdV after the optimization of key reaction parameters. The assay exhibited excellent specificity, sensitivity, and repeatability. The application of the assay was validated by testing 1211 clinical samples, demonstrating coincidence rates ≥ 98.18% with the reference methods. Therefore, a triplex RT-cdPCR was successfully established for the simultaneous and accurate detection of ALV, CIAV, and FAdV.

## 1. Introduction

Avian leukosis virus (ALV) belongs to the genus *Alpharetrovirus* within the family *Retroviridae*, possessing a 7.2–7.8 kb RNA genome. ALV infection can lead to tumors, growth retardation, suboptimal feed conversion ratios, and reduced egg production and hatchability in chickens, directly impacting the economic benefits of the poultry industry [1]. Based on the properties of the envelope glycoprotein, ALV is divided into 11 subgroups, from subgroup A (ALV-A) to ALV-K [1]. Among them, six subgroups (A, B, C, D, J, and K) are exogenous viruses that infect chickens, while ALV-E is an endogenous virus that is typically non-pathogenic or weakly pathogenic [1]. Although ALV has largely been eradicated in international breeding companies, it continues to infect many local chicken breeds in China [2,3,4], and A, B, J, and K are the predominant circulating subgroups [5,6,7]. An epidemiological survey of ALV in diseased chickens and other susceptible animals in China during 2010–2024 revealed an average positivity rate of 25.35%. ALV-A, ALV-B, ALV-J, and ALV-K were all detected, with ALV-J showing the highest prevalence rate [7]. At present, there is no effective vaccine available to prevent ALV, and the clinical symptoms of infected chickens are usually atypical, making them difficult to identify in clinical diagnosis. Therefore, the primary strategy for population eradication is the detection and culling of positive individuals.

Chicken infectious anemia virus (CIAV) belongs to the genus *Gyrovirus* in the family *Anelloviridae*. It is a single-stranded, negative-sense, and non-enveloped DNA virus that possesses a circular genome of approximately 2.3 kb comprising three partially overlapping open reading frames (ORFs), which encode viral proteins VP1, VP2, and VP3, respectively [8]. Caused by CIAV, chicken infectious anemia (CIA) represents an immunosuppressive condition of viral origin. In infected chicks, the hallmark features include aplastic anemia and pronounced depletion of lymphoid cells in the thymus and bone marrow, whereas adult chickens typically show subclinical infection, with reduced egg production, poor growth performance, and immunosuppression. Consequently, this immunosuppression reduces vaccine efficacy and predisposes birds to secondary infections [9]. The discovery of CIAV can be traced back to a vaccine safety incident in Japan in 1974, and it has since been widely detected in chicken flocks worldwide [10,11,12]. CIAV has a high prevalence rate in China and has shown a total positivity rate of 32.9% in southern China from 2016 to 2017, with most strains being highly virulent [13]. An investigation into CIAV in Shandong Province from 2020 to 2022 found that the positivity rates ranged from 12.23% to 17.21% and noted a high incidence of mixed infections with other immunosuppressive pathogens [14]. Beyond the direct immunopathological damage caused by viral infection, CIAV also impacts susceptibility to other pathogens and the efficacy of vaccines. When CIAV infection co-occurs with other pathogens, it can not only exacerbate existing clinical symptoms but also significantly diminish the protective effect of vaccines, accelerating disease progression and even leading to host death [15]. Currently, there is no effective treatment for CIA, and only the measures that relieve clinical signs can be applied to infected chickens. Vaccination offers the best combination of efficacy and economy for preventing and controlling CIAV infection. At present, the globally approved vaccines against CIAV consist mainly of live attenuated strains, which pose safety concerns for chicks due to their potential to revert to virulence [16]. Therefore, the development of reliable pathogen detection methods is crucial for enhancing flock surveillance and minimizing transmission hazards.

Fowl adenovirus (FAdV) is a double-strand DNA virus with 24–48 kb genome, falling within the *Aviadenovirus* genus of the *Adenoviridae* family, and infects all avian species [17]. Most FAdV strains can replicate in healthy birds, often resulting in subclinical infections, and may act as opportunistic pathogens under conditions of immunosuppression [18]. Some FAdV strains are primary pathogens, causing complex clinical symptoms that are difficult to treat. Additionally, FAdV infections are characterized by rapid onset and swift transmission, leading to high mortality rates in infected flocks. Based on differences in group-specific antigens, FAdV strains are classified into three groups [19]. Group I fowl adenovirus (FAdV I) is mainly isolated from the respiratory tract and liver of chickens, ducks, and geese. FAdV II, known as turkey hemorrhagic enteritis virus (TuHEV), is predominantly isolated from turkey lymphocytes and hepatic tissues. The infected birds often present with hemorrhagic enteritis and marbled spleen. FAdV III consists solely of the Egg drop syndrome virus (EDSV). Restriction endonuclease analysis and serum cross-neutralization tests serve as the basis for dividing FAdV I into five species (A to E) and 12 serotypes (FAdV-1 to -8a and FAdV-8b to -11). FAdV I is responsible for acute avian infectious diseases primarily characterized by inclusion body hepatitis (IBH) and hydropericardium syndrome. IBH can be caused by multiple serotypes of FAdV I, but is mainly induced by FAdV-11, FAdV-8a, and FAdV-8b. It primarily affects broilers aged 3 to 8 weeks and is characterized by hepatomegaly, necrosis, and eosinophilic intranuclear inclusion bodies [20]. Since 2015, widespread outbreaks of IBH caused by serotypes 8b and 11, have occurred in Henan, Shandong, Hebei, Jiangsu, Jilin, and other provinces in China, posing a severe threat to the poultry industry [21]. The disease is distinguished by rapid onset and high mortality, often transmitted via vertical and horizontal routes, and its severity can be exacerbated by environmental stress or secondary infections. Immunosuppression resulting from bursal dysfunction can predispose birds to FAdV-induced IBH. Furthermore, when flocks are co-infected with CIAV and ALV along with adenovirus, the morbidity and mortality rates of IBH can increase [22]. It is vital to differentially detect the pathogens for accurate diagnosis. 

Digital PCR (dPCR), as a third-generation PCR technology, is a novel nucleic acid quantification method developed from conventional PCR and quantitative real-time PCR (qPCR). Currently, the application of dPCR technology is rapidly expanding beyond traditional detection fields and has permeated various branches of life sciences, such as clinical diagnosis, genetic testing, and environmental monitoring. The core principle of dPCR technology lies in the miniaturization of a macroscopic PCR system to the nanoliter scale and its distribution into numerous physically isolated reaction units, enabling precise absolute quantification [23]. Through microdroplet or microwell array technology, nucleic acid molecules in a sample are randomly distributed into thousands of physically isolated reaction units, forming a “single-molecule reaction system” [24]. After amplification, the results of positive and negative fluorescent signals from each reaction unit are analyzed using the Poisson distribution to calculate the template copy number in the original sample, thereby enabling highly sensitive detection [25]. Based on differences in reaction partitioning, dPCR technologies can be classified into droplet digital PCR and chip digital PCR. The droplet digital PCR generates water-in-oil droplets based on the immiscibility of oil and water, whereas the chip digital PCR relies on microfabrication techniques to form microchannels and microchambers on a chip, thereby generating an array of microreaction chambers [26]. Compared with qPCR, dPCR technology can directly count the number of target molecules. It does not require a standard curve or rely on a threshold cycle value for analysis; it achieves absolute quantification solely through single-molecule counting [27]. Consequently, it offers considerable advantages and, to some extent, addresses the limitation of qPCR. Following viral infection, many animals present with mild or no clinical signs, thereby making definitive diagnosis difficult during the early stage of infection. Due to its high specificity and sensitivity, dPCR technology is advantageous for applications such as viral load determination, virus subtype identification, and viral mutation detection in viral diseases. For instance, dPCR assays have been developed for specific, sensitive, and accurate detection of African swine fever virus (ASFV) [28,29], Group A porcine rotavirus (PoRVA) [30], Atypical porcine pestivirus (APPV) [31], Bovine leukemia virus (BLV) [32], and Goose astrovirus [33].

In this study, ALV, CIAV, and FAdV were selected as the detection targets for the triplex dPCR assay based on the following considerations. ALV, CIAV, and FAdV are important pathogens currently threatening the poultry industry. These viruses induce different kinds of tumors or subclinical signs. More importantly, they share the critical feature of immunosuppression, which can lead to decreased immunity in flocks, impaired vaccine response, and increased susceptibility to secondary pathogens, resulting in substantial economic losses [1,34]. Furthermore, co-infections with immunosuppressive viruses are common in poultry and can even lead to exacerbated disease outcomes [6], and co-infections of ALV, CIAV, and/or FAdV have been confirmed in clinical cases [35,36,37,38]. Nevertheless, to date, no dPCR assay has been developed to detect and distinguish these three viruses in a single reaction. Therefore, establishing a triplex RT-dPCR for the simultaneous and differential detection of ALV, CIAV, and FAdV could provide rapid and sensitive diagnostic and monitoring tools for these pathogens. In this study, crystal digital PCR (cdPCR) was developed using the Naica™ digital PCR system (Stilla Technologies, Villejuif, France), representing a novel dPCR technology. It combines the advantages of droplet dPCR and chip dPCR by partitioning samples into a two-dimensional droplet array on a single chip through a microchannel network. The droplets are then subjected to on-chip amplification, followed by fluorescence signal detection [39].

## 2. Materials and Methods

### 2.1. Animal Ethics Statement

This study was approved by the Guangxi Center for Animal Disease Control and Prevention (No. 2020-A-02) on 5 November 2020. This study was conducted in accordance with local legislation and institutional requirements. Written informed consent was obtained from the chicken owners.

### 2.2. Reference Viruses

The vaccine strains were obtained from different companies: Muscovy duck reovirus (MDRV, strain CA) and duck tembusu virus (DTMUV, strain FX2010-180P) from Qingdao Yebio Biological Engineering Co., Ltd. (Qingdao, China); Newcastle disease virus (NDV, strain A-VII, strain LaSota), Marek’s disease virus (MDV, strain 814), avian influenza virus (AIV, type H5N1, clade 2.3.4.4h), infectious bronchitis virus (IBV, strain H120), and infectious Bursal disease virus (IBDV, strain B87) from Harbin Pharmaceutical Group Bio-Vaccine Co., Ltd. (Harbin, China). Among these, NDV (strain A-VII) and AIV (Type H5N1) were derived from inactivated vaccines, and the remaining strains were derived from live attenuated vaccines. Positive tissue samples of ALV, CIAV, and FAdV were provided by our laboratory.

### 2.3. Clinical Samples

From February 2025 to April 2026, a total of 1211 clinical samples were collected from 25 breeder chicken farms in Guangxi Zhuang Autonomous Region, China. These samples included tissues (heart, liver, spleen, and kidney), serum, and meconium (collected from newly hatched chicks within 2 h post-hatch, before feed intake). The information on the selected 25 breeder chicken farms is shown in Supplementary Table S1.

### 2.4. Primers and Probes

The specific primers and probes of ALV, CIAV, and FAdV used in this study were synthesized according to the quadruplex RT-qPCR previously developed in our laboratory [38]. The sequence information on primers and TaqMan probes is shown in Table 1.

### 2.5. Extraction of Nucleic Acids

The heart, liver, spleen, and kidney were collected, and approximately 0.1 g of each tissue sample was placed into a 2.0 mL tube that contained 1.0 mL of phosphate-buffered saline (PBS, pH7.2) and two 3 mm steel beads. The tissues were homogenized using a tissue grinder (Retsch MM400, Retsch, Haan, Germany) for 5 min. Approximately 0.3 g meconium was put into a 2.0 mL tube containing 1.0 mL PBS (PH7.2) and vortexed for 1 min. After centrifugation (12,000× *g* for 2 min at 4 °C), 200 μL of the supernatant (from tissue and meconium) was collected for the extraction of total DNA/RNA on a GeneRotex 96 system (TIANLONG, Xi’an, China) using a Nucleic Acid Extraction and Purification Kit (ZIJIAN, Shenzhen, China), following the manufacturer’s protocol. Two hundred μL of sera was collected to extract the total DNA/RNA directly.

### 2.6. Preparation of RNA/DNA Standards

The following kits for constructing the RNA and DNA standards were obtained from TaKaRa Biotechnology Co., Ltd. (Dalian, China): MiniBEST Viral RNA/DNA Extraction Kit, PrimeScript II 1st Strand cDNA Synthesis Kit, PrimeScript™ One Step RT-PCR Kit, In Vitro Transcription T7 Kit, SteadyPure RNA Purification Kit, MiniBEST DNA Fragment Purification Kit, pMD18-T vector, *E. coli* DH5α competent cells, and MiniBEST Plasmid Extraction Kit.

The RNA standard for ALV and the DNA standards for CIAV and FAdV were constructed according to the procedures reported by Mo et al. [38] with minor modification. Briefly, the total nucleic acid was extracted from an ALV-positive sample, transcribed to cDNA, and used as a template to amplify the target fragment. The PCR products were purified and used as a template to amplify RNA in vitro transcription. The products were treated with DNase I and purified. The obtained RNA was called sr-ALV and used as the RNA standard for ALV. Its concentration was measured using a spectrophotometer and calculated using the following formula:

$$Copies/\mu L=\frac{6.02\times 10^{23}\times RNA\;concentration\left(ng/\mu L\right)\times 10^{-9}}{Transcript\;length\left(nt\right)\times 340}$$

The total nucleic acid was extracted from CIAV- or FAdV-positive samples and used as templates to amplify the target fragments. The PCR products were purified, ligated to the pMD18-T vector, and transformed into *E. coli* DH5α competent cells. The positive colonies were selected and expanded in solution culture. The recombinant plasmids were extracted, named sd-CIAV and sd-FAdV, and used as DNA standards for CIAV and FAdV, respectively. Their concentrations were measured and calculated using the following formula:

$$Copies/\mu L=\frac{6.02\times 10^{23}\times plasmid\;concentration\left(ng/\mu L\right)\times 10^{-9}}{plasmid\;length\left(bp\right)\times 660}$$

### 2.7. Optimization of the Reaction Conditions

The primer/probe concentrations, annealing temperatures, and reaction cycles for ALV, CIAV, and FAdV were optimized using the Naica™ digital PCR system (Stilla Technologies, Villejuif, France). The triplex RT-cdPCR reaction system (25 μL) contained 12.5 μL of 2× ApHS One Step RT-PCR MasterMix (Aper Biotechnology, Suzhou, China); 2.5 μL of Fluorescein Sodium Salt (1 μM) (Aper Biotechnology, Suzhou, China); primers and probes for ALV, CIAV, and FAdV at final reaction concentrations ranging from 600 to 1000 nM and from 300 to 500 nM, respectively; 2.5 μL of template; and nuclease-free distilled water to a final volume of 25 μL. The amplification procedure was as follows: 50 °C for 30 min, 95 °C for 5 min; followed by 50 cycles of 95 °C for 15 s, 60 °C for 30 s, and 72 °C for 30 s.

### 2.8. Construction of the Standard Curves

The RNA standard sr-ALV and DNA standards sd-CIAV and sd-FAdV were mixed at a 1:1:1 ratio and 10-fold serially diluted to final reaction concentrations of 1.00 × 10^4^ to 1.00 × 10^0^ copies/μL in the reaction system. The optimized reaction conditions were used to perform triplex RT-cdPCR, and the standard curves for this assay were generated. The linearity of the dPCR assay was assessed by plotting the measured concentrations against the expected concentrations, and the slope and R^2^ values close to 1 indicated excellent quantitative accuracy over the tested dynamic range.

### 2.9. Specificity Assessment

To evaluate whether the established method exhibits cross-reactivity with other viruses, multiplex RT-cdPCR amplification was performed using the following templates: (i) the standard mixture of sr-ALV, sd-CIAV, and sd-FAdV; (ii) the nucleic acids of ALV, CIAV, and FAdV from the clinical positive samples; and (iii) the RNA or DNA of NDV, MDV, AIV, DTMUV, MDRV, IBV, and IBDV from vaccine strains. The nucleic acids from the clinical negative sample and nuclease-free distilled water were served as negative controls.

### 2.10. Sensitivity Assessment

An equal mixture of sr-ALV, sd-CIAV, and sd-FAdV standards was 10-fold serially diluted to final reaction concentration: 1.00 × 10^4^–1.00 × 10^−2^ copies/μL, and triplex RT-cdPCR was carried out to evaluate sensitivity using Poisson distribution analysis.

In addition, the mixture of sr-ALV, sd-CIAV, and sd-FAdV standards with 1.00 × 10^2^ copies/μL was then 2-fold serially diluted, and the concentrations of 0.49–15.63 copies/reaction (20 replicates each concentration) were used as templates. The number of positive replicates was recorded, and Probit regression analysis was used to model the relationship between the detection rate and template concentration for sensitivity assessment.

### 2.11. Repeatability Assessment

The standard mixture of sr-ALV, sd-CIAV, and sd-FAdV was 10-fold serially diluted to 1.00 × 10^4^, 1.00 × 10^3^, and 1.00 × 10^2^ copies/μL of final reaction concentration. Intra-assay repeatability was assessed in triplicate per concentration, and inter-assay reproducibility was assessed on three different days. Repeatability was evaluated by calculating the intra- and inter-assay coefficient of variation (CV).

### 2.12. Detection of Clinical Samples

To evaluate the clinical performance of the established triplex RT-cdPCR method, 1211 clinical samples from Guangxi Zhuang Autonomous Region were tested using three methods: the developed triplex RT-cdPCR, a multiplex RT-qPCR previously established in our laboratory [38], and the reference qPCR methods. The reference qPCR methods consisted of three separate assays: the published qPCR assays for ALV [40], FAdV [41], and CIAV based on the China Agricultural Industry Standard “Diagnostic Techniques for Chicken Infectious Anemia” (NY/T 1187-2019) [42] (https://std.samr.gov.cn/hb/search/stdHBDetailed?id=A7B31164793F3AA4E05397BE0A0A2AA6, accessed on 19 July 2026). SPSS 27.0 was used to analyze coincidence rates and Kappa values for the developed assay against the reference methods to assess diagnostic agreement.

## 3. Results

### 3.1. Construction of RNA/DNA Standards

The ALV *pol* gene was amplified, purified, and used as the template to synthetize RNA in vitro transcription. The obtained RNAs were labeled sr-ALV. Similarly, the CIAV *VP3* and FAdV *Hexon* genes were amplified, purified, and used as templates to construct plasmids. The obtained recombinant plasmids were labeled sd-CIAV, and sd-FAdV, respectively. sr-ALV was used as the RNA standard for ALV, and sd-CIAV and sd-FAdV were used as the DNA standards for CIAV and FAdV, respectively. Subsequently, the concentrations of these three standards were measured using a spectrophotometer, diluted to 1.00 × 10^6^ copies/μL, and stored at −80 °C until use.

### 3.2. Determination of Optimal Reaction Conditions

**Figure 1:**
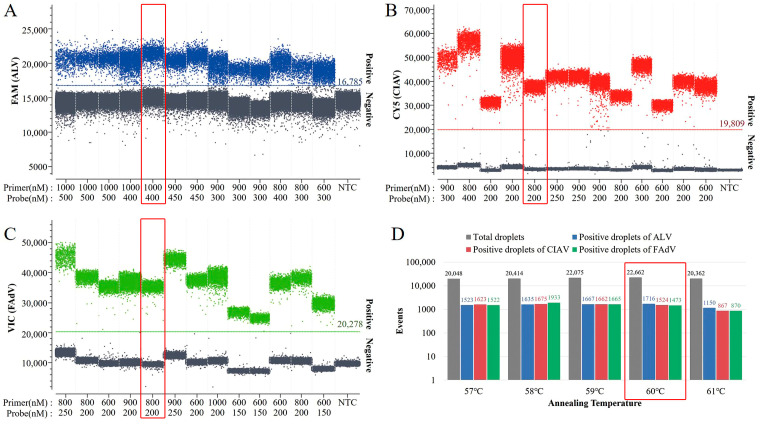
Optimization of the triplex RT-cdPCR reaction conditions. The optimization results of the primer and probe concentrations for ALV (**A**), CIAV (**B**), and FAdV (**C**) and their annealing temperature (**D**) are shown. The red boxes indicate the selected optimal conditions.

The reaction conditions for the triplex RT-cdPCR were optimized using the mixed standards of sr-ALV, sd-CIAV, and sd-FAdV at a final concentration of 1.0 × 10^3^ copies/μL. The optimal concentrations of primers and probes and optimal annealing temperature were determined based on the following criteria: high fluorescence signal intensity of positive droplets, concentrated droplet distribution, clear separation between negative and positive droplets, high absolute concentration, and a low number of dispersed droplets (Figure 1). The optimal reaction system is shown in Table 2. The reaction procedure was as follows: 50 °C 30 min, 95 °C for 5 min; 50 cycles of 95 °C 15 s, 60 °C 30 s, and 72 °C 30 s.

### 3.3. Generation of the Standard Curves

**Figure 2:**
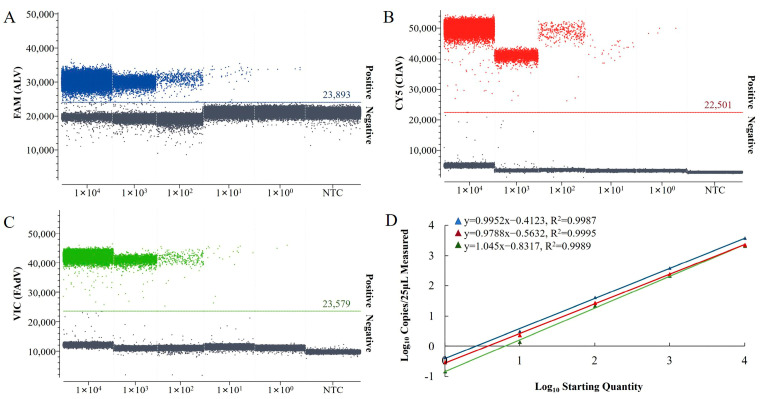
Generation of the triplex RT-cdPCR standard curves. The amplification patterns of sr-ALV (**A**), sd-CIAV (**B**), and sd-FAdV (**C**) at different concentrations and the generated standard curves (**D**) are shown.

The diluted standard mixture of sr-ALV, sd-CIAV, and sd-FAdV with final reaction concentrations of 1.00 × 10^4^ to 1.00 × 10^0^ copies/μL was used as the template for RT-cdPCR amplification to generate the standard curves. The results showed that the slope and correlation coefficient (R^2^) of the standard curves were 0.9952 and 0.9987 for ALV, 0.9788 and 0.9995 for CIAV, and 1.045 and 0.9989 for FAdV, respectively (Figure 2).

### 3.4. Specificity

**Figure 3:**
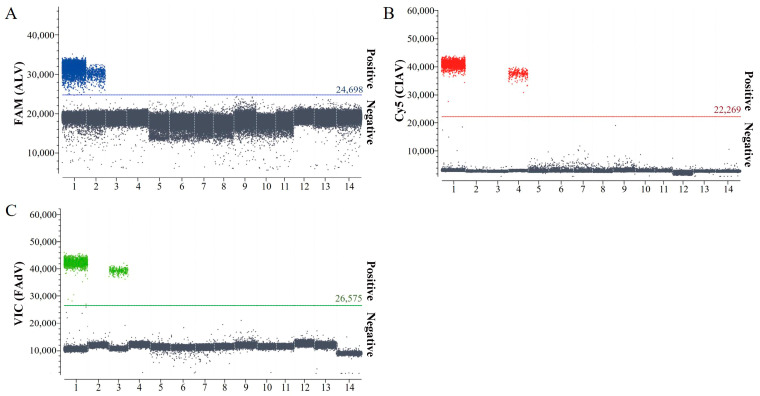
Specificity analysis of the triplex RT-cdPCR. Amplification results of ALV (**A**), CIAV (**B**), and FAdV (**C**) in the FAM, CY5, and VIC channels, respectively. 1: The mixture of sr-ALV, sd-CIAV, and sd-FAdV; 2–4: the clinical positive samples of ALV, FAdV, and CIAV; 5: NDV strain A-VII; 6: NDV strain LaSota; 7: MDV strain 814; 8: AIV type H5N1, clade 2.3.4.4h; 9: DTMUV strain FX2010-180P; 10: MDRV strain CA; 11: IBV strain H120; 12: IBDV strain B87; 13: the clinical negative sample; 14: nuclease-free distilled water.

To assess the specificity of the triplex RT-cdPCR method, the following viral nucleic acids were used as templates to be tested: ALV, CIAV, FAdV, NDV, IBV, MDV, AIV, DTMUV, MDRV, and IBDV. The results showed that the established assay specifically detected only ALV, CIAV, and FAdV, without cross-reactivity with other avian pathogens (Figure 3).

### 3.5. Sensitivity

**Figure 4:**
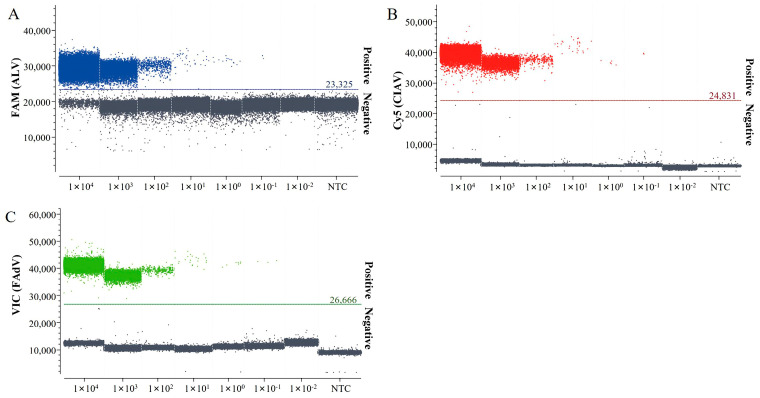
Sensitivity analysis of the triplex RT-cdPCR based on Poisson distribution analysis. Amplification plots of sr-ALV (**A**), sd-CIAV (**B**), and sd-FAdV (**C**) at final reaction concentrations ranging from 1.00 × 10^4^ to 1.00 × 10^−2^ copies/μL. NTC: no-template control using nuclease-free distilled water as the template.

The serially diluted standard mixture from 1.00 × 10^4^ to 1.00 × 10^−2^ copies/μL was used to evaluate the limits of detection (LODs) of the triplex RT-cdPCR based on Poisson distribution analysis. The LODs for sr-ALV, sd-CIAV, and sd-FAdV were determined as 3.75, 3.75, and 5.50 copies/reaction, respectively (Figure 4).

**Figure 5:**
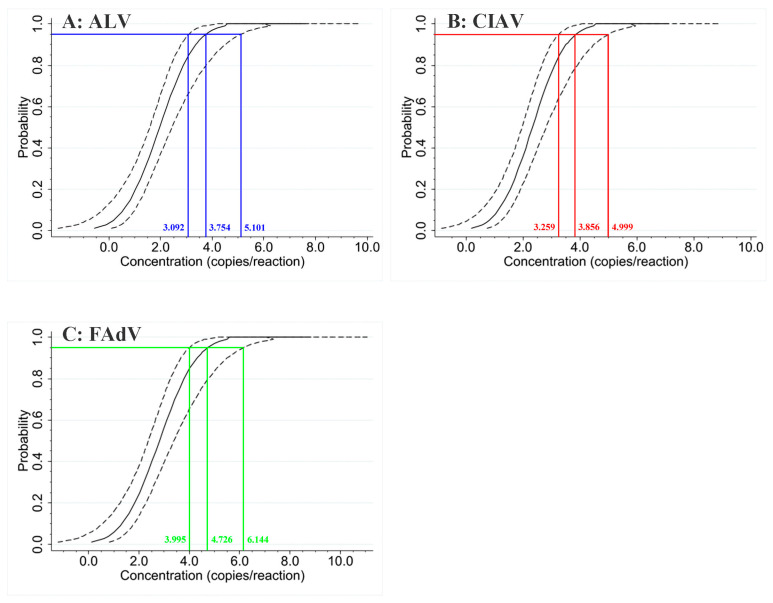
Sensitivity analysis of the triplex RT-cdPCR based on Probit regression analysis. The limits of detection (LODs) for sr-ALV (**A**), sd-CIAV (**B**), and sd-FAdV (**C**) were determined to be 3.754 (95% CI: 3.092–5.101), 3.856 (95% CI: 3.259–4.999), and 4.726 (95% CI: 3.995–6.144) copies/reaction at a 95% confidence level (CI).The solid curve represents the fitted regression line, and the two dashed curves indicate the upper and lower limits of the 95% CI for the fitted curve.

Additionally, the serially diluted standard mixture from 15.63 to 0.49 copies/reaction was used to assess the LODs of the triplex RT-cdPCR based on Probit regression analysis. The positive detection rates are shown in Table 3. The LODs for sr-ALV, sd-CIAV, and sd-FAdV were determined as 3.754 (95% CI: 3.092–5.101), 3.856 (95% CI: 3.259–4.999), and 4.726 (95% CI: 3.995–6.144) copies/reaction, respectively (Figure 5).

### 3.6. Repeatability

The repeatability of the triplex RT-cdPCR method was assessed using three concentrations of 1.00 × 10^4^, 1.00 × 10^3^, and 1.00 × 10^2^ copies/μL of the sr-ALV, sd-CIAV, and sd-FAdV mixture. The results indicated CVs of 0.38–1.77% for intra-assay and 0.31–3.02% for inter-assay (Table 4).

### 3.7. Test Results of the Clinical Samples

**Figure 6:**
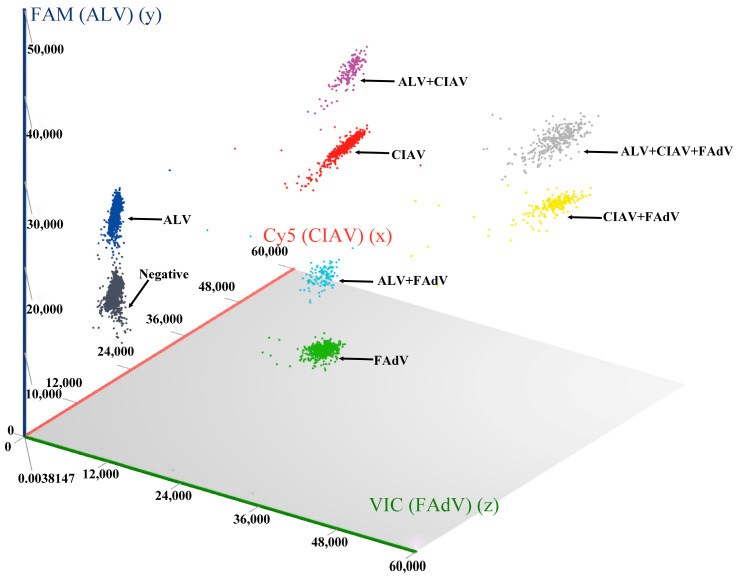
Three-dimensional RT-cdPCR scatter plots of the positive samples. Droplets are plotted based on their fluorescence intensities in the FAM (ALV), Cy5 (CIAV), and VIC (FAdV) channels. Color-coded populations indicate droplets positive for single, dual, or triple targets. Negative droplets are shown in gray.

The 1211 clinical samples collected from Guangxi Zhuang Autonomous Region were tested using the triplex RT-cdPCR method established in this study. The positivity rates for ALV, CIAV, and FAdV were 45.58% (552/1211), 10.73% (130/1211), and 5.20% (63/1211), respectively (Table 5). The detection results are shown in three-dimensional scatter plots in Figure 6. Meanwhile, the 1211 samples were tested using the multiplex RT-qPCR method previously established in our laboratory, and other reference qPCR methods. The results were analyzed with SPSS 27.0 to calculate the coincidence rates, Kappa values (Table 6). The developed triplex RT-cdPCR and the reference methods showed coincidence rates ≥ 98.18%, with Kappa values ≥ 0.94. Regarding the sample types, ALV and CIAV showed the highest detection rates in tissue samples, followed by serum and meconium. In contrast, the detection rates for FAdV followed a different order: serum, meconium, and tissue (Table 7).

## 4. Discussion

China has implemented an avian leukosis (AL) eradication program in breeder flocks since 2008 and has achieved great progress [7]. However, the diversity of chicken breeds and the complexity of infection status in China have led to significant difficulty in AL eradication [43,44,45]. Based on the genetic features of the *VP1* gene, CIAV strains fall into four primary lineages (I, II, III, and IV) [15,34,46]. Following infection, CIAV DNA persists in the host as a covalently closed circular molecule, a feature that complicates its clearance [47,48]. In recent years, FAdV has continuously evolved under immune selection pressure, with strains exhibiting enhanced pathogenicity [49], thereby triggering large-scale outbreaks that seriously threaten the poultry industry [50,51,52]. Therefore, ALV, CIAV, and FAdV are still widely prevalent in chicken flocks in China. Nowadays, ALV and CIAV are significant immunosuppressive pathogens, and co-infection with these two viruses commonly occurs, significantly accelerating disease progression and greatly increasing poultry mortality [36,53,54]. FAdV also exhibits immunosuppressive properties, further raising the risk of mixed infections [35,55]. The clinical manifestations and pathological features of infections caused by these three viruses are similar, posing challenges for diagnosis and differentiation. Currently, although the singleplex dPCR detection methods have been reported for CIAV and FAdV [56,57], no dPCR method capable of simultaneously detecting ALV, CIAV, and FAdV has been reported. To address this gap, a triplex RT-cdPCR method targeting the *pol* gene of ALV, the *VP3* gene of CIAV, and the *Hexon* gene of FAdV was established in this study for the simultaneous detection of these three viruses, aiming to provide technical support for breeder farms to formulate precise monitoring strategies.

In this study, specific primers and probes were designed and utilized to achieve accurate detection of ALV, CIAV, and FAdV without cross-reactivity with other pathogens, conferring high specificity to the developed assay. The concentration ratios of the primers and probes were optimized to maintain balanced fluorescence signals across the three channels. Two analysis approaches (Poisson distribution and Probit regression) were used to assess the sensitivity of the RT-cdPCR assay. First, ten-fold serial dilutions of the RNA/DNA standards were subjected to triplex RT-cdPCR, and the LODs were calculated using Poisson distribution analysis, yielding values of 3.75, 3.75, and 5.50 copies per reaction for ALV, CIAV, and FAdV, respectively. In addition, serial dilutions were assessed using Probit regression analysis, revealing LODs of 3.754, 3.856, and 4.726 copies per reaction for ALV, CIAV, and FAdV, respectively. The LODs derived from Poisson distribution analysis represent a theoretical estimate based on single-molecule partitioning, reflecting the intrinsic detection capability of the method [58]. In contrast, the LODs obtained via Probit regression analysis were determined through serial dilution experiments, a method that models the relationship between detection probability and template concentration [59]. This approach provides a realistic sensitivity threshold that can be stably and reproducibly achieved under practical testing conditions. The combination of these two approaches compensates for deviations between theoretical estimates and empirical results, and they can be used to jointly verify the sensitivity of the established assay. The minimal discrepancy observed between the two datasets indicates that the developed RT-cdPCR assay exhibits low systematic error and high robustness. Mo et al. established a multiplex RT-qPCR assay for the detection of ALV, CIAV, FAdV, and avian reovirus (ARV), with LODs of 136.66, 129.59, 139.79, and 133.20 copies/reaction, respectively [38]. This indicates that the developed triplex RT-cdPCR assay demonstrated about 30-fold higher sensitivity than the corresponding multiplex RT-qPCR assay. In addition, according to previous studies, intra- and inter-assay CVs below 10% are considered acceptable [60]. To assess the repeatability of the developed triplex RT-cdPCR assay, three concentrations of sr-ALV, sd-CIAV, and sd-FAdV (1.00 × 10^4^, 1.00 × 10^3^, and 1.00 × 10^2^ copies/μL) were used to determine the CVs. The results demonstrate that the intra- and inter-assay CVs were less than 1.77% and 3.02% in this study, respectively, indicating that the developed assay exhibited high repeatability and stability.

The 1211 clinical samples from 14 cities across Guangxi Zhuang Autonomous Region, China, were tested using the established triplex RT-cdPCR assay. The results demonstrated that the positivity rates of ALV, CIAV, and FAdV were 45.58%, 10.73%, and 5.20%, respectively. These data indicated that ALV had the highest positivity rate, which might be attributed to the absence of obvious clinical symptoms in ALV-infected chickens, leading to a lack of attention to AL eradication in breeder farms. As for co-infection, the highest co-infection rate was observed for ALV and CIAV (5.53%), followed by triple co-infection (3.80%). This finding was consistent with previous reports showing that co-infection with immunosuppressive viruses is common in poultry flocks and increases susceptibility to other pathogens [6,36,53,55]. Compared with the reference methods, the developed assay showed coincidence rates above 98.18% and Kappa values exceeding 0.94. These results indicate substantial agreement between these methods, confirming the reliability of the established method. In addition, different sample matrices, including tissue, serum, and meconium, were used to detect ALV, CIAV, and FAdV in order to assess the application of the developed assay. The results confirm that tissue, serum, and meconium are suitable for the detection of these viruses and verify that the assay could use these sample matrices to test ALV, CIAV, and FAdV, which further validates the broad applicability of the established method.

The triplex RT-cdPCR assay developed here enables the simultaneous detection of ALV, CIAV, and FAdV, with high sensitivity and specificity, offering substantial application value in breeder farm monitoring, vaccine quality control, and the prevention and control of these three diseases. In terms of pathogen monitoring and eradication in breeder farms, all three pathogens can be vertically and horizontally transmitted, and their mixed infections are common [1,36,54,61], making it difficult to comprehensively assess risks using single-pathogen detection. The developed method allows for the simultaneous differentiation of these three viruses within one reaction, raising eradication monitoring efficiency and furnishing technical support for selective culling and source purification. ALV, CIAV, and FAdV might pollute chicken embryo-derived live vaccines, such as those for Marek’s disease (MD), and Newcastle disease (ND), and can be introduced into chicken flocks, leading to immunization failure or disease transmission [46,62,63,64]. The developed triplex RT-cdPCR can be used for the simultaneous screening of three exogenous viruses in vaccines, preventing the contamination of vaccines with ALV, CIAV, and FAdV. Therefore, the developed method can be used to detect these pathogens for clinical diagnosis, as well as for vaccine quality control.

Evidently, dPCR technology still has some disadvantages at the present stage. Although it provides benefits such as high sensitivity and absolute quantification, it still faces limitations in practical application, including limited throughput, high costs, and complex operation. The instrumentation and consumables are expensive, making it difficult to widely promote at the grassroots level. Additionally, the droplet generation and transfer steps require high operational stability, and the throughput is insufficient to meet the demands of large-scale screening [65]. In the future, with the continuous advancement of microfluidics, fully automated platforms, and multicolor fluorescence sorting technologies, dPCR is expected to achieve breakthroughs in cost reduction, throughput improvement, and enhanced multiplexing capabilities, positioning itself as an important technical tool for disease eradication in breeder farms and vaccine quality control. At present, the development of multiplex dPCR assay to detect several targets in one reaction is a good choice for decreasing the cost of this technology.

## 5. Conclusions

A triplex RT-cdPCR assay for concurrent detection and differentiation of ALV, CIAV, and FAdV was successfully established in this study. This assay exhibited strong specificity, high sensitivity, and excellent repeatability. It allowed for the rapid and reliable detection of very low concentrations of ALV, CIAV, and FAdV in the clinical samples in a single reaction within 2 h, offering a robust technical tool for the rapid and accurate detection of these viruses.

## Figures and Tables

**Table 1:** Primers and probes for the triplex RT-cdPCR assay.

Virus	Gene	Primer/Probe	Sequence (5′→3′)	Product (bp)
ALV	*pol*	ALV-F	TAGCTGATTTGGGGGCAAG	145
		ALV-R	ACTCCCTCCTGTCCCAT	
		ALV-P	FAM-GTGGCCATGGCACTTCTGCTGT-BHQ1	
CIAV	*VP3*	CIAV-F	GAAGATACTCCACCCGGAC	92
		CIAV-R	CCAATCCGGATCTCTCTGC	
		CIAV-P	CY5-CACCAACAAGTTCACGGCCGTT-BHQ2	
FAdV	*Hexon*	FAdV-F	GCCTACCCGCAATGTCACT	120
		FAdV-R	CTGTCCCCCACGTTTAAGC	
		FAdV-P	VIC-TATCCCACCCAGACGGACGACAC-BHQ1	

**Table 2:** The optimal reaction system of the triplex RT-cdPCR assay.

Regent	Volume (μL)	Final Concentration (nM)
ApHS One Step RT-PCR MasterMix (2×)	12.5	1×
Fluorescein Sodium Salt (1 μM)	2.5	100
ALV-F (25 μM)	1	1000
ALV-R (25 μM)	1	1000
ALV-P (25 μM)	0.4	400
CIAV-F (25 μM)	0.8	800
CIAV-R (25 μM)	0.8	800
CIAV-P (25 μM)	0.2	200
FAdV-F (25 μM)	0.8	800
FAdV-R (25 μM)	0.8	800
FAdV-P (25 μM)	0.2	200
Total nucleic acids	2.5	/
Nuclease-free distilled water	Up to 25	/

**Table 3:** The positivity rates of 2-fold serial dilution of the RNA/DNA standards.

Standard	Concentration (Copies/Reaction)	Number of Samples	Positive Samples	Detection Rates (%)
sr-ALV	15.63	20	20	100
	7.82	20	20	100
	3.91	20	19	95
	1.96	20	10	50
	0.98	20	6	30
	0.49	20	0	0
sd-CIAV	15.63	20	20	100
	7.82	20	20	100
	3.91	20	19	95
	1.96	20	7	35
	0.98	20	2	10
	0.49	20	0	0
sd-FAdV	15.63	20	20	100
	7.82	20	20	100
	3.91	20	16	80
	1.96	20	6	30
	0.98	20	1	5
	0.49	20	0	0

**Table 4:** Repeatability analysis of the triplex RT-cdPCR assay.

Standard	Concentration (Copies/μL)	Intra-Assay for Repeatability			Inter-Assay for Reproducibility		
		$\bar{X}$ (Copies/Reaction)	SD	CV (%)	$\bar{X}$ (Copies/Reaction)	SD	CV (%)
sr-ALV	1.00 × 10^4^	95,875.00	535.41	0.56	97,419.44	1669.18	1.71
	1.00 × 10^3^	9285.00	138.26	1.49	9211.39	52.19	0.57
	1.00 × 10^2^	1015.00	7.07	0.70	989.72	17.90	1.81
sd-CIAV	1.00 × 10^4^	55,833.33	246.08	0.44	56,194.44	619.79	1.10
	1.00 × 10^3^	5992.50	39.74	0.66	6012.50	18.71	0.31
	1.00 × 10^2^	675.00	4.08	0.60	656.94	12.81	1.95
sd-FAdV	1.00 × 10^4^	51,175.00	194.72	0.38	51,677.78	770.71	1.49
	1.00 × 10^3^	5338.33	72.61	1.36	5366.11	19.83	0.37
	1.00 × 10^2^	527.50	9.35	1.77	550.83	16.62	3.02

**Table 5:** Detection results of the triplex RT-cdPCR for the clinical samples.

Region	Number	Number of Positive Samples						
		ALV	CIAV	FAdV	ALV + CIAV	ALV + FAdV	CIAV + FAdV	ALV + CIAV + FAdV
Wuzhou	92	32	15	12	4	1	3	8
Yulin	214	96	25	20	8	3	0	17
Baise	53	28	19	5	8	0	0	5
Liuzhou	45	24	8	6	1	1	0	5
Fanchenggang	30	19	12	9	4	2	1	6
Hechi	146	44	18	3	14	0	0	3
Guigang	39	20	4	0	2	0	0	0
Qinzhou	60	33	0	0	0	0	0	0
Guilin	185	90	17	3	16	2	0	0
Nanning	90	43	4	3	3	2	0	1
Beihai	90	46	1	1	0	0	0	1
Hezhou	90	39	2	0	2	0	0	0
Laibin	45	20	1	0	1	0	0	0
Chongzuo	32	18	4	1	4	1	0	0
Total	1211	552	130	63	67	12	4	46
Positivity rate (%)		45.58	10.73	5.20	5.53	0.99	0.33	3.80

**Table 6:** The coincidence of the developed RT-cdPCR and the reference qPCR assays.

Pathogen	Positive Samples			Coincidence Rate		Kappa Value	
	RT-cdPCR	RT-qPCR	qPCR	RT-qPCR	qPCR	RT-qPCR	qPCR
ALV	552/1211	540/1211	530/1211	99.01% (98.28–99.43%)	98.18% (97.26–98.80%)	0.98	0.96
CIAV	130/1211	126/1211	117/1211	99.67% (99.15–99.87%)	98.93% (98.17–99.37%)	0.98	0.94
FAdV	63/1211	59/1211	56/1211	99.67% (99.15–99.87%)	99.42% (98.81–99.72%)	0.97	0.94

**Table 7:** Detection results of ALV, CIAV, and FAdV in different types of samples using the developed triplex RT-cdPCR.

Sample Type	Positive Samples		
	ALV	CIAV	FAdV
Tissue	152/229 (66.38%)	51/229 (22.27%)	4/229 (1.75%)
Serum	226/481 (46.99%)	49/481 (10.19%)	35/481 (7.28%)
Meconium	174/501 (34.73%)	30/501 (5.99%)	24/501 (4.79%)
Total	552/1211 (45.58%)	130/1211 (10.73%)	63/1211 (5.20%)

## Data Availability

The data and materials that support the findings of this study are available from the corresponding author upon reasonable request.

## Supplementary Materials

The following supporting information can be downloaded at: https://www.mdpi.com/article/10.3390/ani16142269/s1. Table S1. The information on the clinical samples collected from 25 breeder farms.
