# Supplementary material for: A Triplex Crystal Digital RT-PCR for the Detection of Avian Leukosis Virus, Chicken Infectious Anemia Virus, and Fowl Adenovirus
Source: Animals (Basel). 2026 Jul 22;16(14):2269. doi: 10.3390/ani16142269 (PMC13404849; doi:10.3390/ani16142269)
Supplement: Supplementary file 1 [file animals-16-02269-s001.zip › animals-4401355-supplementary.pdf]

Supplementary Materials

Table S1 The information on the clinical samples collected from 25 breeder farms.

Farm	Geographic region	Sampling date	Breed	Age(days)	Total samples	Tissue	Serum	Meconium
1	Wuzhou	Jun, 2025	Guangxi Yellow Chicken	128; 130; 1	92	12	40	40
2	Yulin	Feb, 2025	Guangxi Yellow Chicken	110	5	5	0	0
3	Yulin	Jun, 2025	Xiayan Chicken	100	29	29	0	0
4	Yulin	Jun, 2025	Guangxi Yellow Chicken	150; 1	80	0	30	50
5	Yulin	Feb, 2026	Guangxi Yellow Chicken	90; 240; 1	100	20	30	50
6	Baise	Jul, 2025	Guangxi Yellow Chicken	120; 110	53	23	30	0
7	Liuzhou	Aug, 2025	Guangxi Yellow Chicken	180	30	0	30	0
8	Liuzhou	Apr, 2026	Guangxi Yellow Chicken	120	15	0	15	0
9	Fangchenggang	Sep, 2025	Guangxi Yellow Chicken	130	30	0	30	0
10	Hechi	Mar, 2025	Guangxi Yellow Chicken	180; 1	80	0	30	50
11	Hechi	Mar, 2025	NYao Chicken	160	5	5	0	0
12	Hechi	May, 2025	Qibainong Chicken	1	61	0	0	61
13	Guigang	Nov, 2025	Guangxi Yellow Chicken	100	19	19	0	0
14	Guigang	Dec, 2025	Guangxi Yellow Chicken	115	20	20	0	0
15	Qinzhou	Dec, 2025	Guangxi Partridge Chicken	112	30	30	0	0
16	Qinzhou	Dec, 2026	Guangxi Partridge Chicken	150	30	0	30	0
17	Guilin	Jan, 2026	Guangxi Yellow Chicken	160; 100; 1	95	15	30	50
18	Guilin	Jan, 2026	Guangxi Yellow Chicken	120; 160; 1	90	10	30	50
19	Nanning	Feb, 2026	Guangxi Partridge Chicken	287; 330; 1	90	10	30	50
20	Beihai	Feb, 2026	Guangxi Partridge Chicken	207; 207; 1	90	10	30	50
21	Hezhou	Feb, 2026	Guangxi Partridge Chicken	56; 201; 1	90	10	30	50

22	Laibin	Mar, 2026	Guangxi Yellow Chicken	135	30	0	30	0
23	Laibin	Apr, 2026	Guangxi Yellow Chicken	122	15	0	15	0
24	Chongzuo	Jul, 2025	Guangxi Yellow Chicken	150	11	11	0	0
25	Chongzuo	Apr, 2026	Guangxi Yellow Chicken	150	21	0	21	0
**Total**					**1211**	**229**	**481**	**501**

Note: Vaccination against ALV, CIAV and FAdV using commercial vaccines is not performed in the 25 selected farms.
